# Quantification of the Upper Extremity Motor Functions of Stroke Patients Using a Smart Nine-Hole Peg Tester

**DOI:** 10.1155/2018/7425858

**Published:** 2018-04-10

**Authors:** Ákos Jobbágy, Anikó Rita Marik, Gábor Fazekas

**Affiliations:** ^1^Department of Measurement and Information Systems, Budapest University of Technology and Economics, POB 91, Budapest 1521, Hungary; ^2^National Institute for Medical Rehabilitation, Szanatórium Street, Budapest 1121, Hungary; ^3^Szent János Hospital, Diós Árok 1, Budapest 1125, Hungary

## Abstract

This paper introduces a smart nine-hole peg tester (s-9HPT), which comprises a standard nine-hole peg test pegboard, but with light-emitting diodes (LEDs) next to each hole. The s-9HPT still supports the traditional nine-hole peg test operating mode, in which the order of the peg placement and removal can be freely chosen. Considering this, the s-9HPT was used in lab research to analyze the traditional procedure and possible new procedures. As this analysis required subjects with similar levels of dexterity, measurement data from 16 healthy subjects (seven females, nine males, 25–80 years old) were used. We consequently found that illuminating the LEDs in various patterns facilitated guided tests of diverse complexity levels. Next, to demonstrate the clinical application of the s-9HPT, the improvement in the hand dexterity of 12 hospitalized stroke patients (45–80 years old, six females and six males) was monitored during their rehabilitation. Here, we used traditional and guided tests validated by healthy subjects. Consequently, improvements were found to be patient specific. At the beginning of rehabilitation, traditional tests suitably indicate improvements, while guided tests are beneficial following improvements in motor functions. Further, the guided tests motivated certain patients, meaning the rehabilitation was more effective for these individuals.

## 1. Introduction

The nine-hole peg test was introduced in 1971 [1], together with a mechanical tool for measuring finger dexterity [2, 3]; further, upon its introduction, specific dimensions were provided for both the board and the pegs used. The traditional test (TRT) is quite simple, and healthy persons can complete it in 15–20 seconds. To perform it, the subject must, using only one hand, pick nine pegs up one at a time from the holder and place them in the holes in the board in arbitrary order. When all nine pegs are in the holes, they must then be removed, also one at a time, and with the same hand. The result of the test is the time that has elapsed from the moment the subject picked up the first peg to the moment they returned the last peg to the holder or placed it on the table. The execution time must be measured by a person supervising the test.

Assessments of hand movement do not rate fine motor skills; instead, they are rather functional tests. By analyzing the results of such tests, medical doctors, or physiotherapists can diagnose the severity of the disorder in question and the level of self-management possible and can estimate the prospective improvement. In short, the objective assessment of fine motor function greatly assists effective therapy.

The nine-hole peg test has been extensively studied in previous literature. For instance, several prior studies have tested its effectiveness with patients with various illnesses; examples of such studies are as follows: Heller et al. [4] found the nine-hole peg test to be suitable for rating the dexterity of patients recovering from acute strokes; Earhart et al. [5] tested 262 patients with Parkinson's disease and found the result of the nine-hole peg test to be a clinically useful measure for assessing their upper extremity function; and Feys et al. [6] found the nine-hole peg test to be reliable and applicable for assessing multiple sclerosis patients with different levels of upper limb impairment. Furthermore, the nine-hole peg test has also been applied in other related studies: Brunner et al. [7] and Paquin et al. [8] used the nine-hole peg test to assess the functional recovery of stroke patients; Mathiowetz et al. [9] and Wang et al. [10] published adult norms for the nine-hole peg test; Wade [11] found that dexterity disability is best assessed using the nine-hole peg test or the ten-hole peg test; Wang et al. [12] recommended the nine-hole peg test for inclusion in the motor battery of the NIH Toolbox; and Koyama et al. [13] measured thumb and index finger distance during the nine-hole peg test and found the placement phase to be more informative than the removal phase.

We evaluated medical doctors' experiences concerning the traditional nine-hole peg test and, as a result, the limitations of the low-tech pegboard became evident. Consequently, a smart nine-hole peg tester (s-9HPT) was developed at the Department of Measurement and Information Systems, Budapest University of Technology and Economics. In this paper, we begin by analyzing both the traditional and guided tests based on recordings of healthy subjects using the smart device. Then, we demonstrate the clinical applicability of the s-9HPT by having stroke survivors perform traditional and guided tests that have been validated by the healthy subjects.

## 2. Materials and Methods

As mentioned in the previous section, the main aim of this research was to analyze the nine-hole peg test procedure using the s-9HPT; additionally, however, a further aim was to assess the clinical applicability of the new tests facilitated by s-9HPT.

### 2.1. Tested Persons

Twelve hospitalized stroke patients (Table 1) and 16 healthy control subjects (seven females and nine males, aged 25–80 years, all but one being right-handed) were tested. All subjects provided written informed consent. The research was performed in accordance with the Declaration of Helsinki, and the study protocol was approved by the Scientific and Research Ethics Committee of Szent János Hospital Budapest (protocol no: 001/2016). Stroke patients were selected from the patients of the Department of Physical and Rehabilitation Medicine of Szent János Hospital, Budapest. Specifically, the inclusion criteria for recruitment were (1) upper extremity functional impairment due to the damage of the central nervous system and (2) aged between 18 and 85 years. Meanwhile, the exclusion criteria were (1) plegia of the upper extremities that rendered motor functions untestable, (2) the presence of any disorder influencing hand function that is not related to the central nervous system, and (3) full legal incapacity or partial legal capacity. Patients' actual functional states were assessed for both hands at hospitalization and at the end of the rehabilitation program (i.e., when they were discharged from the hospital) using the Functional Independence Measure (FIM, [14, 15]) (this was performed by a physician who specialized in physical and rehabilitation medicine) and the Barthel Index (BI) (taken by a nurse). Stroke patients repeated the test session on several occasions during their hospitalization period. The minimum number of tests performed by a patient was five, and the maximum was 16 (with the average being nine).

FIM and BI were also used to reveal changes in the patients' functional ability; these measures are widely used to assess stroke patients during rehabilitation. Specifically, FIM features 18 items within six domains and focuses on both activities of daily living and cognition, while BI features 10 items but only assesses activities of daily living.

In regard to the healthy control group, the inclusion criterion was: aged between 18 and 85 years; concurrently, the exclusion criterion was the presence of any disease influencing hand function. The hand dominance of the tested persons was determined by the therapists.

### 2.2. s-9HPT

**Figure 1:**
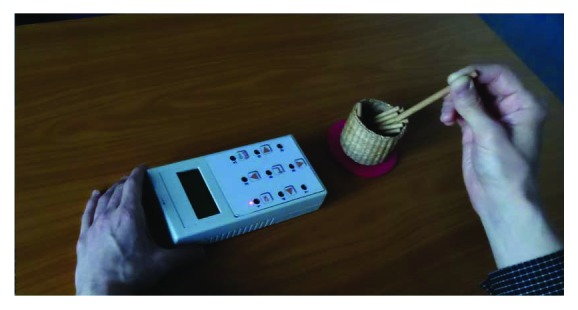
The s-9HPT and the peg holder aligned for a right-handed person.

Using the same mechanical dimensions as the traditional pegboard for the nine-hole peg test, the s-9HPT (Figure 1) has several extra functions. The most obvious of these is that the test can be completed without the presence of a supervisor, as it automatically measures users' execution times. Such unassisted usage is helped by the incorporation of a delayed start to the test: when the “start” button is pressed, the device displays the following messages in sequence for one second each: “ready”–“steady”–“go.” At the moment the “go” message appears, a buzzer also sounds. Then, two measurements of execution time are performed. The first ranges from the time the buzzer sounds to the removal of the last peg, while the second begins with the insertion of the first peg and ends with the removal of the last one. The difference between the two measurements is the time taken to pick up the first peg and insert it into a hole.

The s-9HPT has a sensor in each hole that allows it to detect the presence of a peg. The device measures not only the time required to complete the test but also the time intervals between consecutive peg insertions and removals. Thus, the device affords the measuring of three phases of the total execution time:

- Placement time: ranging from the insertion of the first peg until the insertion of the ninth peg
- Intermediate time: ranging from the insertion of the ninth peg until the removal of the first peg
- Removal time: ranging from the removal of the first peg until the removal of the ninth peg

On the device, a light emitting diode (LED) is located next to each hole; in Figure 1, it can be seen that the LED corresponding to hole 1 is illuminated. By adding a visual component, guided tests (GUTs) can be performed. An example of such a test would involve a subject being asked to insert a peg into a hole with a corresponding illuminated LED; when this peg is correctly inserted, the LED at this hole remains lighted and the LED for the next hole in the sequence illuminates. When the ninth peg is placed in the hole, all LEDs are turned off and on for a short period (0.2 s), and then all but one LEDs are switched off. At this point, the peg associated with the illuminated LED should then be removed.

There is another test that diverges slightly from this process: the random test (RAT). Here, the device might request that the subject, while in the process of inserting pegs, remove a peg before all nine have been inserted. During RATs, an unlit LED, while all other eight LEDs are illuminated, is used to signify that the corresponding peg should be removed.

Considering the above descriptions, it is clear that this system affords the setting of GUTs with differing levels of complexity. The TRT does not specify the order the pegs should be placed into and removed from holes; thus, a subject can choose different orders every time he/she is tested, causing low reproducibility. GUTs eliminate this uncertainty. Further, the order of placement and removal can be programmed beforehand by the tester. Moreover, as in both GUTs and RATs subjects are required to perceive and recognize lit and unlit LEDs, as well as place pegs in corresponding holes, it should be noted that deteriorated cognitive function results in longer execution time.

### 2.3. The Measurement Procedure

In this study, the same medical doctor supervised all tests. To begin, the doctor informed each subject of the correct method of using the s-9HPT, allowing them to become acquainted with the device before commencing the assessment. The tester was placed on the table in front of the test subject, and each user kept the tester in a fixed position using the hand that was not being tested. Meanwhile, the pegs were placed in a holder next to the tester, on the same side as the hand being tested. As mentioned earlier, the pegs were to be picked up one at a time, either from the holder or from the holes; if a peg fell onto the table, the participant picked it up and continued the test; if a peg fell to the floor, the supervisor picked it up and placed it back in the holder. When the participant was comfortable with the device, the supervisor began a TRT; this entailed the insertion of nine pegs into the holes in an arbitrary order, and then the removal of each peg, also in an arbitrary order.

The device was rotated by 90 degrees so that the display was closer to the nontested hand; this position guarantees the same level of usage as the traditional nine-hole peg testing device, while improving the visibility of the LEDs. Five GUTs with different complexities were defined; there were two main kinds of GUTs: those entailing a simple sequence of holes, and those featuring a pseudorandom sequence. A test sequence could be made more difficult by requesting that the test subject place pegs into holes that are located behind pegs (from the angle of the tested hand) that have already been placed in holes. Specifically, for the GUTs, the following hole orders were used (for an s-9HPT that has been rotated counterclockwise, as in Figure 1, and with the subject using their right hand):

GUT 1 (easy):          3 6 9 2 5 8 1 4 7

GUT 2 (intermediate):       7 4 1 8 5 2 9 6 3

GUT 3 (difficult):        9 6 8 3 5 7 2 4 1

GUT 4 (pseudorandom, easy):    6 1 4 3 2 7 5 9 8

GUT 5 (pseudorandom, difficult):   8 1 4 3 5 9 2 7 6

Meanwhile, for the RAT, as mentioned above, the placement and removal of pegs was conducted in a random order; further, during such tests, the removal of placed pegs was requested intermittently, at which times the subject would remove pegs they had placed up to that point. A sample random order is the following (“P”: placement, “R”: removal): P1–P5–P3–R5–R1–P9–P8–R8–P2–R3–R9–P4–P1–P6–R1–R4–R2–P8.

#### 2.3.1. Contents of the Test Sessions for Healthy Subjects

1. Getting acquainted with the s-9HPT, signing the information sheet and consent form
    Items (2)–(9) completed with dominant hand
    Items (3)–(8) completed with non-dominant hand.
2. TRT, repeated a number of times (for most subjects, twice) until the subject becomes familiar with the device and the test procedure
3. Three TRTs
4. GUT1
5. GUT2
6. GUT3
7. GUT4
8. GUT5
9. RAT

The above session plan was identical for all healthy patients; that is, the number of each form of test and the order were not randomized.

#### 2.3.2. Contents of the Test Sessions for Stroke Patients

A different test plan was provided to the stroke patients; this was because the healthy subjects were tested in order to assess the traditional nine-hole peg test procedure and the smart tester, while the stroke patients were tested to show that the s-9HPT gives more information concerning stroke patients' rehabilitation than the simple mechanical nine-hole peg test device. Further, stroke patients became tired within a few minutes of testing, meaning their test sessions had to be shorter.

At the beginning of the first test session involving stroke patients, the subjects were also given an opportunity to familiarize themselves with the s-9HPT, and they signed an information sheet and consent form. Then, they repeatedly performed TRTs until their results stabilized. The remainder of the first test session comprised the following tests:

Items (1)–(4); completed with the non-affected hand

Items (1)–(4); completed with the affected hand

1. TRT, repeated three times
2. GUT1
3. GUT4
4. GUT5

Further test sessions began with three TRTs. Then, for each patient, depending on their actual condition, the test session was either terminated after the third TRT or continued through the application of GUTs, which were given on an approximately weekly basis. Details on the length of each patient's rehabilitation process are provided in Table 1.

### 2.4. Statistical Methods

To perform our analysis, well-known and widely used statistical methods were applied: Pearson's correlation coefficient (PCC), Spearman's rank correlation (SRC), coefficient of variation (COV), the intraclass correlation coefficient (ICC), and a nonparametric rank probe (Kruskal-Wallis). Meanwhile, Excel 2010 (Microsoft [16]) and MATLAB (The Mathworks Inc., Natick, MA USA, version R2007b) were used to calculate these parameters.

Further, the exponential fit of the measured data and the calculation of adjusted R^2^ parameters (characterizing the goodness of the fit) were also performed using MATLAB.

## 3. Results

Overall, over 600 tests were completed by the healthy subjects, while over 1000 were completed by the hospitalized stroke patients. Although all participants were asked to begin the test upon hearing the buzzer, some participants, especially patients, a couple of times commenced the test too early or late. Thus, during the evaluation, the moment the first peg was inserted was set as the start of the test, as this increased the reliability of the results.

Although the test sessions comprised of different test items for stroke patients than for healthy persons, all participants' test results were found to have improved; in some cases, improvement was even found during the same test session, as the participants became accustomed to the device while they were using it.

**Figure 2:**
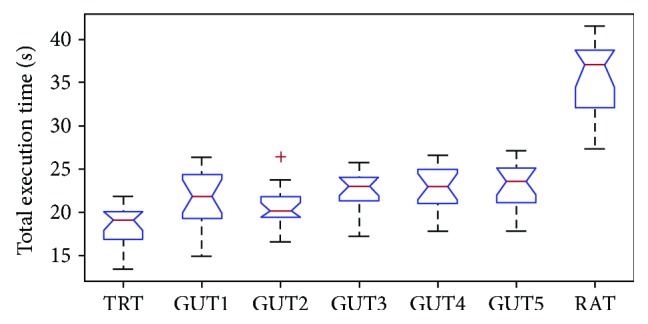
Total execution times of healthy persons using their dominant hand. TRT: traditional test; GUT: guided test; RAT: random test.

For GUT1 and GUT2, the correct orders in which the pegs should be placed were relatively easy for the participants to replicate; meanwhile, during TRTs (i.e., without LED guiding), both patients and healthy persons generally spontaneously placed the pegs in the same order as requested by GUT1. However, GUT3 featured a more sophisticated sequence, while GUT4 and GUT5 required the subjects to pay close attention. GUTs have three levels of complexity. This was determined by using the nonparametric Kruskal-Wallis probe to examine the test sessions (minimum of six) performed by each of the 16 healthy subjects; consequently, three groups (TRT; GUT3, GUT4, and GUT5; and RAT) with significantly different mean ranks were revealed (*α* = 0.05). GUT1 and GUT2 are not significantly different from TRT (PCC values: 0.80, 0.84) or from GUT3 (PCC values: 0.77, 0.75), GUT4 (PCC values: 0.73, 0.86), and GUT5 (PCC values: 0.56, 0.58) but are significantly different from RAT (PCC values: 0.31, 0.33). The results for healthy subjects are summarized in Figure 2 (showing, for subjects' dominant hands, TRT scores and results for GUT1, GUT2, GUT3, GUT4, GUT5, and RAT). A surprising finding was that for most healthy persons, GUT1 required a longer execution time than the more difficult GUT2. However, the reason for this is that the participants initially found the GUT format to be unusual, but grew accustomed to the method as they used it and consequently found the more difficult GUTs performed afterward to be more normal. During the traditional test, the time intervals of the phases had higher COV than the ratios of these time intervals to the total execution times. The results (mean, COV) were as follows: placement time: 12.1 s, 12.3%; removal time: 5.7 s, 12.7%; intermediate time: 1.1 s, 29.3%. Meanwhile, placement-time ratio was 64%, 3.0%; removal-time ratio was 30%, 5.6%; intermediate-time ratio was 6%, 20.3%.

The stroke patients' test results for their use of the s-9HPT are shown in Table 2 (illustrating the average TRT scores, as well as scores for GUT1, GUT4, and GUT5 for the first and last complete test sessions performed in the hospital). Average daily improvement in total execution time was calculated in percent for the TRT using (1), and improvements in GUT values were calculated accordingly.

(1) $$\begin{array}{c} Traditional\;test\;improvement\;\left(\%\right)=\left(1-\sqrt[{n}]{\frac{traditional\;test\;\left(last\;day\right)}{traditional\;test\;\left(first\;day\right)}}\right)*100. \end{array}$$

**Figure 3:**
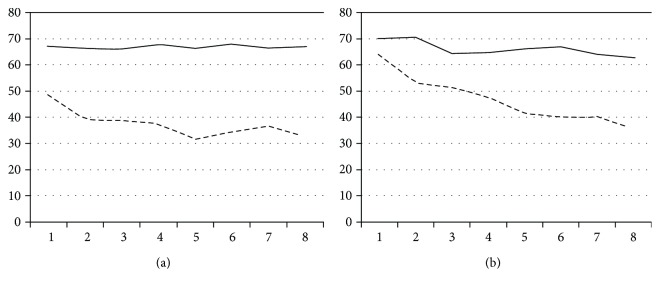
The improvement in total execution time (in se88c, dashed line) and the change in placement-time ratio (in percentage, solid line) during traditional tests for two stroke patients (affected hand) over the course of the rehabilitation, S3 (left) and S4 (right).

However, the stroke patients' results could not be averaged; the patients had different levels of motor disability, and their improvements during rehabilitation also differed. The results of two stroke patients (S3 and S4, affected hand) over the course of the rehabilitation are shown in Figure 3, along with total execution time and the ratio of placement time to total execution time.

Further, results for stroke patients' nonaffected hands were also found to have improved (see Table 2); however, the level of improvement here was much less than that for affected hands. This shows that the noted improvements in affected hands were only slightly based on the practice patients gained through performing the test.

## 4. Discussion

The s-9HPT facilitates guided tests of diverse complexity levels. These tests provide more detailed assessments of hand dexterity than the traditional nine-hole peg test. Nevertheless, guided tests require sound cognitive function. The traditional and guided tests were analyzed based on the results of healthy subjects, and this analysis greatly assisted in the assessment of stroke patients.

Stroke patients do not constitute a homogeneous group; thus, their results cannot be averaged. Each patient's data were analyzed individually. At the beginning of the rehabilitation, TRT was found to be appropriate for assessing patients' hand movements. Guided tests were used to assess fine motor skills and were applied following observations of improvements in motor functions. Our results attest that improvements in stroke patients' motor skills are reflected differently by nine-hole peg test results and functional ability measures (FIM, BI). The correlation between FIM and BI was found to be weak, with the PCC decreasing from 0.58 at the commencement of the rehabilitation to 0.38 at the end. The correlation between BI and NHPT tests was also weak, with PCC being better at the beginning of the rehabilitation (PCC values: BI–TRT: −0.35, BI–GUT1: −0.65, BI–GUT4: −0.41, BI–GUT5: −0.35) than that at the end (PCC values: BI–TR: −0.16, BI–GUT1: −0.18, BI–GUT4: −0.14, BI–GUT5: −0.10). Further, there was no linear relationship between FIM and NHPT tests at the beginning of the rehabilitation (PCC values: FIM–TRT: −0.07, FIM–GUT1: −0.03, FIM–GUT4: 0.01, FIM–GUT5: 0.14) or at the end (PCC values: FIM–TRT: 0.02, FIM–GUT1: 0.09, FIM–GUT4: 0.16, FIM–GUT5: 0.27). However, this discrepancy can be explained by the fact that FIM and BI evaluate functional independence, which differs from hand dexterity.

In general, stroke patients had higher removal-time rates and, thus, lower placement-time rates than healthy persons. This indicates that for stroke patients, making the required hand movements and grabbing pegs are difficult. The level of difference between the difficulty experienced placing a peg into a hole and that involved in placing a peg into the much bigger peg holder was smaller for patients than for the healthy subjects. However, results are person specific; illustrating this point, Figure 3 shows two patients' (S3 and S4) differing rehabilitation progress.

Analyzing the stroke patients' results, a marked improvement was found relating to the number of days spent in hospital. Decreases in the total execution time of the TRTs can be approximated using the *T*(*d*) = *A*∗exp(−*B*∗*d*) + *C* function, where *T* is the total execution time of a TRT; *d* is the time in days from the first test session to the actual test session; and *A*, *B*, and *C* are patient-specific constants.

The goodness of exponential fit to data was tested using MATLAB's curve-fitting tool, and the median of adjusted R^2^ for the 12 stroke patients was found to be 0.67 of the exponential fit. For nine of these patients (S1, S3, S4, S6, S7, and S9–12), the exponential curve fitting was found to be good (for these nine patients, the median of adjusted R^2^ was 0.88 (min.: 0.53, max: 0.99)). Meanwhile, however, for the other three patients, the exponential fit was worse than simply fitting a horizontal line; this caused negative R^2^ values.

Such exponential curve fitting can help therapists decide if further improvements (decreases) in TRT execution time can be expected in the near future. We evaluated the goodness of exponential fit for each stroke patient and the relation between the estimated final values for the fitted exponential functions and the last-measured values for TRT execution time. These data can help determine whether the rehabilitation program is worth continuing. Consequently, considering our participants, the exponential function fit must be rejected for S2, S5, and S8, as the results corresponding to these patients exhibit a fluctuation around a given value; this means that for these patients, continuing the rehabilitation program would not be reasonable. Meanwhile, the exponential fit was good for the other patients' results. In particular, S1, S3, S7, S10, S11, and S12 achieved the estimated final (minimum) value with regard to their total TRT execution time. Thus, the rehabilitation program is not expected to yield further improvement for these participants, either. However, during the last complete test session, for S4, S6, and S9, the total execution time was much longer than the estimated minimum value; consequently, for these patients, the rehabilitation program is expected to yield further improvements in the near future (it should be noted that there are also other aspects that should be taken into account in order to appropriately determine if the rehabilitation program should be terminated or continued). Figure 3 shows the difference between the progress of S3 and S4.

The guided tests mainly confirmed the qualification of the approach based on the exponential fit, with S4 and S10 representing exceptions. For S1, S2, S3, S5, S7, S8, and S12—similar to the healthy subjects—GUT4 and GUT5 required longer execution times than TRT. Further, the difference between the affected and nonaffected hands with regard to GUT4 execution time was a maximum of 10 s.

S6 and S9 differed from all other patients: their execution times in GUT1, GUT4, and GUT5 were shorter than those in TRT, and the average differences between their affected and nonaffected hands were substantially greater than those of the other patients (GUT1: 27.4 and 31.8 s, resp., average of other patients: 6.4 s; GUT4: 19.3 and 18.2 s, resp., average of other patients: 9.1 s; GUT5: 18.5 and 19.7 s, resp., average of other patients: 9.4 s). These results affirm that for S6 and S9, further improvements can soon be expected with regard to the motor function of their affected hands.

Based on the exponential fit, S4 was qualified to continue the rehabilitation. However, similar to the healthy subjects, she produced longer execution times in all guided tests than in TRT, and the difference between her affected and nonaffected hands in TRT, GUT1, and GUT5 was much smaller than the differences shown by S6 and S9. These results do not suggest that further improvement can be expected in the motor function of S4's affected hand in the near future.

For S10, the difference between the affected and nonaffected hands with regard to GUT4 execution time was longer than 10 s (12.8 s), while GUT1, GUT4, and GUT5 execution times were equal. Consequently, his rehabilitation should be continued.

Through GUTs, therapists can personalize test items for a given patient. In our sample, some patients (S4, S7, and S10) mentioned enjoying the GUTs; these patients were motivated to improve (decrease) their total execution times, and this made the rehabilitation more effective. However, there were also patients who disliked (S5) or were almost unable to perform (S9 and S11) the GUTs, and consequently, these tests did not improve the efficiency of their rehabilitation.

Next, it should be noted that at the beginning of the rehabilitation process, S9 and S11 stated that they found the GUTs to be too difficult; however, by the end of the program S9 was able to perform GUTs. Unfortunately, as she did not perform a GUT at the beginning of her rehabilitation, her improvement could not be quantified. Further, during the final test session, S2 became confused while performing GUT4 because he was disturbed by a nurse accidentally entering the room. Consequently, the score for this test (48.79 s) was much worse (longer execution time) than that S2 could have achieved, but he was too tired to repeat GUT4 at the end of the test session.

Another finding of our research is that the better a stroke patient's nine-hole peg test score at the beginning of the rehabilitation program, the smaller their improvement at the end (see Table 2). Further, substantial improvement at the beginning of rehabilitation (S3, S4, S9, S10, and S12) was mainly due to improvements in arm (and not in hand) movement. For patients' affected hands, the correlation between the result of the TRT given at the commencement of the program and the difference between the test scores for the TRTs at the beginning and at the end of the rehabilitation program was excellent: PCC = 0.996 and SRC = 0.944. Further, the correlation between the average TRT result during the first complete hospital test session and the average daily improvement was also excellent: PCC = 0.92 and SRC = 0.92.

Test-retest reliability [17] was rated based on the three TRTs performed by the 16 healthy persons at the beginning of their test sessions. The ICC for the 16 data groups was calculated using Excel, and the results are convincing: for the dominant hand, ICC was 0.921 (95% confidence interval: 0.82–0.97), while for the nondominant hand, ICC was 0.918 (95% confidence interval: 0.80–0.97).

## 5. Study Limitations

In total, the 12 stroke patients and the 16 healthy control persons completed over 1600 tests, and we found this to be sufficient to analyze the traditional nine-hole peg test, to verify the evaluation algorithms and the GUTs, and to give feedback to engineers developing the s-9HPT. However, further tests involving more stroke patients are required to validate the clinical applicability of the s-9HPT. In addition, further tests are needed to evaluate the diagnostic power of the detailed time values the s-9HPT measures; specifically: placement, intermediate, and removal time as well as time intervals between consecutive peg placements and removals.

## 6. Conclusions

The TRT results for the healthy persons were categorized into three age groups, and these are presented in Table 3; these data are similar to norms published in previous related studies [9, 12, 18].

Finally, in accordance with the findings of Wang et al. [10], Earhart et al. [5], and Oxford Grice et al. [18], within the healthy group, we found that women performed slightly better than men (see Table 3).

The nine-hole peg test is considered an effective test for hand dexterity, as it can objectively qualify and quantify the progress of (e.g., Parkinson's) or recovery from (e.g., stroke) certain diseases. However, the s-9HPT provides more detailed information on hand dexterity. Using the s-9HPT, therapists can personalize test sessions for patients, selecting traditional and guided tests of different complexities; moreover, the results measured by the s-9HPT can help therapists decide if the rehabilitation program can be expected to yield further improvements for a patient. Meanwhile, guided tests motivate some patients, making their rehabilitation more effective; further, there are marked differences in stroke patients' progress, meaning each patient's data must be analyzed individually. Finally, the s-9HPT can be used by the patients themselves at home without the presence of a supervisor; such home application can help the rehabilitation of stroke patients.

## Figures and Tables

**Table 1:** Tested stroke patients.

Identity	Age	Sex	Affected/dominant hand	Type of stroke: ischemic (I) or hemorrhagic (H)	Days from stroke to hospitalization	Days from stroke to first test session	Days spent in hospital	Number of test sessions	Barthel index, %		FIM motor scores	
									At hospitalization	At discharge from hospital	At hospitalization	At discharge from hospital
S1	71	M	R/R	I	19	53	59	8	85	100	58	83
S2	65	M	L/R	I	19	26	21	7	95	100	58	81
S3	70	F	L/R	I	36	41	21	8	85	100	83	97
S4	80	F	L/R	I	26	30	27	8	60	95	35	89
S5	74	F	R/R	I	10	14	32	9	75	90	58	81
S6	65	F	R/R	I	56	78	42	9	70	80	50	78
S7	45	M	L/R	I	8	29	46	12	75	100	64	83
S8	76	M	R/R	H	20	27	18	8	90	100	81	86
S9	64	F	L/R	I	20	23	23	8	75	100	67	86
S10	69	M	R/R	H	18	32	29	5	55	100	36	81
S11	66	F	R/R	I	23	37	73	16	25	95	33	78
S12	70	M	R/L	I	11	12	35	10	35	90	53	86

**Table 2:** Results for hospitalized stroke patients. This shows, in seconds, total execution times for the first and last complete test sessions.

	S1	S2	S3	S4	S5	S6	S7	S8	S9	S10	S11	S12
Days between first and last complete test sessions	15	8	16	14	23	14	25	22	15	14	20	32
*Affected hand, total execution time in s*												
Average of TRTs, first complete test session	29.07	31.86	47.29	63.68	27.85	70.49	66.32	44.33	153.13	79.61	90.3	72.67
Average of TRTs, last complete test session	22.46	29.60	32.55	35.63	21.12	49.19	37.08	34.78	63.21	32.5	62.0	36.66
GUT1, first test session	30.85	32.88	46.03	70.46	30.91	56.4	67.39	43.53	—	67.01	—	69.27
GUT1, last test session	23.07	26.75	33.9	37.19	30.08	43.44	31.75	34.06	61.64	36.75	—	39.08
GUT4, first test session	46.56	35.87	48.04	74.85	34.59	57.41	70.1	47.46	—	66.97	—	68.4
GUT4, last test session	31.77	32.42	36.46	46.72	27.11	45.98	40.13	36.67	59.74	36.99	—	46.09
GUT5, first test session	32.36	35.61	52.24	86.27	37.42	62.16	101.51	53.1	—	69.15	—	67.01
GUT5, last test session	29.89	31.72	44.52	38.78	27.21	45.9	48.36	38.31	61.51	36.98	—	56.84
*Nonaffected hand, total execution time in s*												
Average of TRTs, first complete test session	25.33	31.6	24.6	25.71	26.29	23.9	26.27	29.56	40.69	27.24	28.5	45.88
Average of TRTs, last complete test session	23.72	27.44	24.91	20.98	21	21.76	25.57	26.7	31.4	22.53	24.5	28.16
GUT1, first test session	25.29	31.26	25.99	35.01	32.03	26.13	26.13	32.35	46.86	25.53	—	43.81
GUT1, last test session	25.38	26.25	26.63	24.19	26.68	23.3	26.2	28.72	34.65	23.34	—	33.09
GUT4, first test session	33.13	39.99	38.78	36.84	37.26	28.09	26.36	42.83	47.94	26.03	—	49.45
GUT4, last test session	27.44	—	29.29	25.33	30.8	26.67	30.11	32.46	41.54	24.22	—	36.44
GUT5, first test session	32.78	32.07	36.44	37.3	32.8	28.09	29.68	34.59	47.02	26.15	—	52.57
GUT5, last test session	27.34	27.87	33.55	27.77	26.75	27.45	28.86	42.32	41.22	24.42	—	37.24
*Average daily improvement, in percent*												
TRT, affected hand	1.71	0.92	2.31	4.06	1.20	2.54	2.30	1.10	5.73	6.20	1.86	2.12
GUT1, affected hand	1.92	2.55	1.89	4.46	0.12	1.85	2.97	1.11	n.a.	4.20	n.a.	1.77
GUT4, affected hand	2.52	1.26	1.71	3.31	1.05	1.57	2.21	1.17	n.a.	4.15	n.a.	1.23
GUT5, affected hand	0.53	1.44	0.99	5.55	1.38	2.14	2.92	1.47	n.a.	4.37	n.a.	0.51
TRT, nonaffected hand	0.44	1.75	−0.08	1.44	0.97	0.67	0.11	0.46	1.71	1.35	0.74	1.51
GUT1, nonaffected hand	−0.02	2.16	−0.15	2.61	0.79	0.82	−0.01	0.54	1.99	0.64	n.a.	0.87
GUT4, nonaffected hand	1.25	n.a.	1.74	2.64	0.82	0.37	−0.53	1.25	0.95	0.51	n.a.	0.95
GUT5, nonaffected hand	1.20	1.74	0.52	2.09	0.88	0.16	0.11	−0.92	0.87	0.49	n.a.	1.07

**Table 3:** Traditional test results for the healthy participants.

	Tested subjects	Average (s)	Std. dev. (s)
Age group	21–35 (7 persons)	17.94	1.13
	36–55 (3 persons)	17.99	1.37
	61–80 (5 persons)	19.62	1.22
Sex	All (16 persons)	18.48	1.22
	Females (7 persons)	18.18	1.87
	Males (9 persons)	18.71	2.13
